# Safety of the Immediate Reconstitution of Poly‐l‐Lactic Acid for Facial and Body Treatment—A Multicenter Retrospective Study

**DOI:** 10.1111/jocd.16560

**Published:** 2024-09-17

**Authors:** Roberta Vasconcelos‐Berg, Julia Real, Franziska Wenz, Luiz Eduardo Toledo Avelar

**Affiliations:** ^1^ Margarethenklinik University Hospital of Basel Basel Switzerland; ^2^ Private Practice Belo Horizonte Brazil; ^3^ Department of Forensic Anthropology Civil Police Department Belo Horizonte Minas Gerais Brazil

**Keywords:** biostimulator, dilution, filler, immediate reconstitution, PLLA‐SCA, poly‐l‐lactic acid, reconstitution, safety

## Abstract

**Background:**

Poly‐l‐lactic acid (PLLA‐SCA; Sculptra) was approved in 1999 in Europe and 2004 in United States as a collagen biostimulator. It is a freeze‐dried preparation containing 150 mg PLLA‐SCA per vial and, since approval, has been recommended to be reconstituted 72 h before treatment, which can hinder its use in clinical practice. In 2021, the manufacturer authorized the reconstitution of PLLA‐SCA immediately before use.

**Objective:**

To evaluate adverse events in patients treated with immediately reconstituted PLLA‐SCA on the face, body, and scars.

**Method:**

This was a retrospective analysis of medical records of patients treated with immediately reconstituted PLLA‐SCA for aesthetic purposes from January 1, 2021, to December 31, 2021, at two medical centers.

**Results:**

A total of 274 treatment sessions were conducted on 167 patients (ranging from 1 to 5 sessions per patient). Of these, 228 sessions (151 patients) targeted the face, 39 sessions (22 patients) addressed the body, and 7 sessions (5 patients) focused on scars. The mean final concentration of PLLA‐SCA was 15.30 mg/mL for the face, 8.35 mg/mL for the body, and 10.53 mg/mL for scars. The majority of injections were administered with a blunt cannula (face: 87.3%, body: 100%, scars: 57%), and in 6 out of 7 scar treatments, PLLA‐SCA was additionally applied topically after fractional treatment. One patient developed a PLLA‐SCA nodule 30 days after facial treatment, which resolved after two saline injections. The most common adverse events were bruising (face: 6.57%, body: 7.69%) and mild pain (face: 3.07%). No events required further intervention.

**Conclusion:**

This study reports an adverse event profile with immediately reconstituted PLLA‐SCA, used on the face, body, and scars, similar to that reported with PLLA‐SCA reconstituted 72 h prior to use.

**Trial Registration:**

This was a retrospective study of medical records at two medical centers, and trial registration was not required.

## 1 Introduction

Poly‐l‐lactic acid (PLLA‐SCA; Sculptra; Galderma, Sweden) is a collagen biostimulator first approved for aesthetic use in Europe in 1999 and the United States in 2004 for the restoration and correction of the signs associated with HIV‐associated lipoatrophy, which includes fat loss of the limbs, buttocks, and face, and in 2009, for correction of nasolabial folds and other facial wrinkles. Initially, the main indication was volume restoration in patients with facial lipodystrophy [1]. Subsequently, the product has also been used for facial rejuvenation, mainly focusing on improving skin laxity and restoring volume [2, 3], and recently received approval for the correction of fine lines and wrinkles in the cheek area [4]. Currently, the substance is also indicated for body treatments in various regions, with a focus on reducing flaccidity, increasing the volume of the gluteal region, and improving skin quality, such as reducing cellulite [5]. As a result of the increase in local collagen production following injection, there are also reports of PLLA‐SCA being injected subcutaneously and/or applied after fractionated technologies (such as laser therapy and microneedling) to reduce the appearance of scars, the so‐called drug delivery technique [6, 7, 8].

Sculptra is formulated as a freeze‐dried preparation containing 150 mg PLLA‐SCA per vial, although the recommended reconstitution volume of the vial has varied considerably since its launch. Initially, it was recommended to reconstitute an ampoule containing 150 mg of PLLA‐SCA with 3 mL of sterile water for injection (SWFI) for facial treatments. However, it was later concluded that high concentrations of PLLA‐SCA increased the incidence of noninflammatory subcutaneous nodules [9] and, for this reason, the volume of dilution recommended by the manufacturer has progressively increased over time. It is currently recommended that PLLA‐SCA should be reconstituted, for facial use, in 8‐mL SWFI plus 1‐mL lidocaine [10]. For PLLA‐SCA use in parts of the body other than the face, it is recommended that this reconstitution volume is increased to 16–20 mL of total solution, including 1 or 2 mL lidocaine [5, 10].

Since PLLA‐SCA first became available it has been recommended that it is reconstituted with SWFI 72 h before use, with lidocaine being added to the vial at the time of treatment. This is because it was believed that the early reconstitution would better hydrate the PLLA‐SCA particles [11]. However, this process can represent a logistical challenge for the injecting physician as it is necessary to schedule the treatment at least 3 days after the first visit and the dilution of PLLA‐SCA needs to be coordinated with the date of the patient's appointment. In addition, the wastage of ampoules may be an inefficiency if the patient does not attend on the day of application.

Recently, Baumann et al. [12] demonstrated that the physicochemical properties of viscosity, concentration of excipients, pH, and particle size of immediately reconstituted PLLA‐SCA remained identical to PLLA‐SCA reconstituted 72 h earlier. In 2021, Bravo and Carvalho [13] analyzed the rate of complications following injection with immediately reconstituted PLLA‐SCA in a total of 58 facial treatments in 26 female patients. For each treatment, the total volume used for reconstitution was 12 mL (10 mL SWFI and 2 mL lidocaine), with a follow‐up period of 90 days. Two patients (3.44%) each developed a subcutaneous nodule within the first month of treatment and had complete regression after intervention with injection of 1.0 mL 0.9% saline solution into the nodule twice with an interval of 1 week and local massage.

The results of these studies indicate that the reconstitution of PLLA‐SCA immediately before treatment does not appear to affect its physicochemical properties compared with reconstitution 72 h prior to use. The clinical safety profile of PLLA‐SCA also seems to be preserved. To date, there have been relatively few published studies involving immediately reconstituted PLLA‐SCA, although in a clinical trial, Palm et al. [14] evaluated the effectiveness of immediately reconstituted PLLA‐SCA in reducing wrinkle severity of nasolabial folds, and the same group also performed a separate medical chart review on immediately reconstituted PLLA‐SCA [15]. In both studies, effectiveness and safety were not compromised when PLLA‐SCA was injected immediately after reconstitution [14, 15]. In addition, the clinical trial evaluating PLLA‐SCA in the treatment of cheek wrinkles (NCT04124692) [16] used immediately reconstituted PLLA‐SCA and reported it to be effective and well tolerated [16]. However, no published safety data are available regards the use of immediately reconstituted PLLA‐SCA in body or scar treatments.

Hence, the aim of this retrospective study was to evaluate any adverse events resulting from the injection of immediately reconstituted PLLA‐SCA for aesthetic purposes in various areas of the face and body and align these with published data from the medical literature for PLLA‐SCA reconstituted 24 to 72 h prior to use.

## 2 Methods

This was a retrospective study performed at two medical centers. The medical records of patients treated with immediately reconstituted PLLA‐SCA (Sculptra, Galderma, Sweden) for aesthetic purposes between January 1, 2021, and December 31, 2021, were included. The following information was obtained from the medical records: age at the time of treatment; sex; number of treatments; total volume and components of PLLA‐SCA reconstitution; total amount (in mg) of injected PLLA‐SCA; treated areas (face, body, or scar); anatomical treatment plan; use of needle or blunt cannula; association of transdermal delivery after a fractional treatment (“drug delivery”); presence of complications; and follow‐up time. These data are presented descriptively.

**FIGURE 1:**
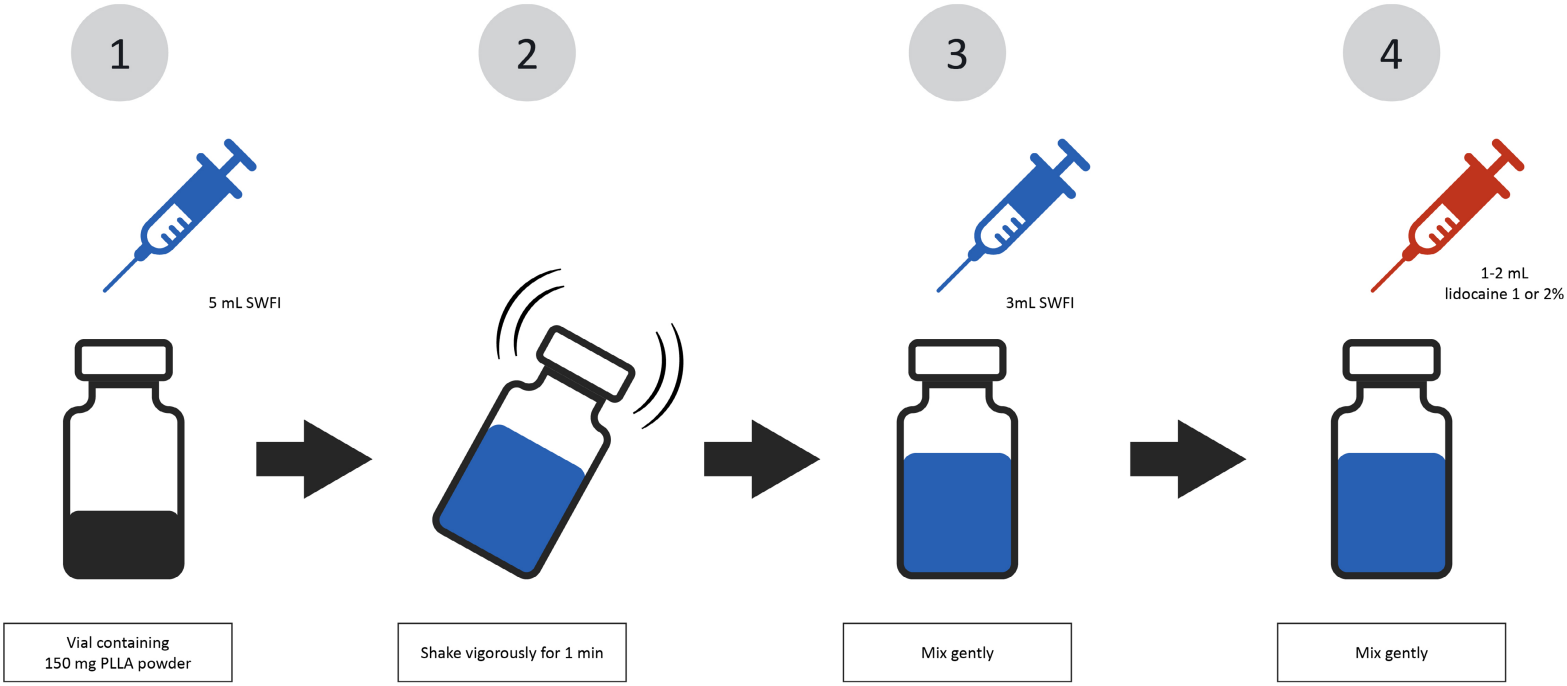
Illustration of the new reconstitution method performed immediately prior to injection of poly‐l‐lactic acid (PLLA‐SCA) treatment. At room temperature, 5 mL of sterile water for injection (SWFI) is injected into the vial (1), followed by immediate vigorous agitation by hand for 1 min (2), followed by the injection of a further 3 mL of SWFI into the vial (3), resulting in an 8 mL solution; 1–2 mL of lidocaine (with or without epinephrine) is then added to the vial (4). This is the final solution recommended by the manufacturer for facial treatments. For body treatments, a further 6–10 mL of SWFI should be added to the facial solution.

The immediate reconstitution of PLLA‐SCA was performed as instructed by the manufacturer: at room temperature, 5 mL of SWFI was injected into the vial, followed by immediate vigorous agitation by hand for 1 min, and then a further 3 mL of SWFI was injected into the vial plus 1 mL of lidocaine with or without epinephrine (Figure 1). For facial treatments, this was the solution used. For body treatments, an additional 6–10 mL of SWFI was added.

Immediately after treatment, the treated area was massaged vigorously and patients were instructed to perform the same massage according to a 5–5–5 rule (5 min, 5 times a day, and for 5 days).

All patients had direct contact with the treatment center to report any adverse events.

The project was analyzed and approved for implementation by the Local Ethics Committee and complied with the laws of the country in which it was performed.

## 3 Results

A total of 167 patients, 152 females aged 29–72 years and 15 males aged 29–69 years, were treated using a total of 311 vials, each containing 150 mg of immediately reconstituted PLLA‐SCA, across 274 treatments for facial and body areas (Table 1), with the goals to improve skin laxity, overall skin quality, or scar appearance. All patients had standardized pictures taken before and after the treatment. The mean follow‐up time after the treatment was 510 days (SD = 100).

**TABLE 1:** Number and percentage of treatment sessions with immediately reconstituted PLLA‐SCA by area and region.

Area	Region	Number of sessions	% of treatments
Face	Total	228	100
	Preauricular	223	98
	Mandibular	223	98
	Temporal	221	97
	Zygomatic	32	14
	Submalar	31	14
	Prejowl	11	5
	Submental	9	4
	Piriform fossa	2	1
Body	Total	39	100
	Buttocks	14	36
	Neck	12	31
	Thighs	8	21
	Abdomen	8	21
	Upper arm	1	3
Scars	Total	7	100
	Shoulders	3	43
	Face	2	29
	Neck	1	14
	Suprapubic	1	14
	Knee	1	14

Abbreviation: PLLA‐SCA, poly‐l‐lactic acid (Sculptra).

The investigating physicians did not note in the medical records an increase in cannula blockage or other technical difficulties during the injection procedure when using the immediately reconstituted PLLA‐SCA compared with their previous personal experiences with PLLA‐SCA reconstituted 24–72 h prior to use.

### 3.1 Facial Rejuvenation Treatment

PLLA‐SCA was used for facial rejuvenation in 151 patients (138 women, 13 men) undergoing a total of 228 treatments (1–5 sessions per patient; mean 1.5, SD 0.8). The mean patient age at the time of treatment was 50.2 years (SD = 10.4; range 29–72 years) (Table 2).

**TABLE 2:** Patient demographics, PLLA‐SCA concentration, treatment techniques utilized, and adverse event rates in patients receiving treatment with immediately reconstituted PLLA‐SCA by region.

	Face	Body	Scar
Demographics			
Number of patients	151	22	5
Number of sessions	228	39	7
Median age (SD), years	49.9 (10.3)	47.0 (8.9)	38.0 (8.4)
Reconstitution and dosage of PLLA‐SCA			
Number of vials of 150 mg PLLA‐SCA per session	0.33 to 2	1 to 5	0.5 to 1
Final volume (range), mL	9–10	16–20	9–20
Final mean concentration of PLLA‐SCA (SD), mg/mL	15.30 (0.64)	8.35 (0.71)	10.53 (4.20)
Treatment technique			
Number of treatments	1–5	1–3	1–2
Injection with blunt cannula, *n* (%)	199 (87.3)	39 (100)	4 (57.1)
Injection with needle, *n* (%)	4 (1.8)	0	1 (14.3)
Injection with blunt cannula + needle, *n* (%)	22 (9.6)	0	2 (28.6)
Drug delivery, *n* (%)	0	0	6 (85.7)
Adverse events, *n* (%)			
All	21 (9.21)	3 (7.69)	0
Bruising	15 (6.57)	3 (7.69)	0
Mild pain	7 (3.07)	0	0
Local edema	1 (0.44)	0	0
Nodule	1 (0.44)	0	0

Abbreviations: PLLA‐SCA, poly‐l‐lactic acid (Sculptra); SD, standard deviation.

Reconstitution of the PLLA‐SCA occurred immediately prior to treatment and was performed according to the manufacturer's current recommendation (Figure 1). The final volume used for facial treatment was between 9 and 10 mL with a final PLLA‐SCA concentration of 15.00–16.66 mg/mL (mean 15.30; SD 0.64). The median dose of PLLA‐SCA administered per treatment session for the entire face was 150 mg (range 50–300 mg) (Table 2).

The regions of the face most frequently treated were the preauricular, mandibular, and temporal regions (Table 1). In 199 treatment sessions, a 22G blunt cannula was used; in four, a 23G needle was used; and in 25 sessions, a 23G needle and a 22G cannula were used in the same patient (Table 2). The application plan was subcutaneous for all regions except for the piriform fossa, where supraperiosteal application was performed.

In 21 sessions (9.21%), the patients reported an adverse event or discomfort after the treatment. The most frequent adverse events were bruising (15 events, 6.57%) and mild pain (7 events, 3.07%) (Table 2); all adverse events required no intervention and were resolved within 1 week of treatment. One patient (0.44%) developed a submental edema 1 day after treatment; this lasted for 3 days and then resolved spontaneously. One patient (0.44%) developed a small subcutaneous nodule above the right mandible angle. The nodule was diagnosed by the physician 30 days after the first treatment. This occurred in a 31‐year‐old female who had been treated with a 150 mg vial of PLLA‐SCA in the temporal, preauricular and mandibular regions. The vial had been diluted with 8 mL of SWFI and 1 mL of lidocaine containing epinephrine. During follow‐up discussion, the patient indicated not performing posttreatment massage to the area as instructed. The nodule was treated with intralesional injection of 2 mL of 0.9% saline using a 30G needle twice within a 7‐day period; the nodule regressed completely 1 week after the second injection.

### 3.2 Body Treatment

PLLA‐SCA was used for body treatment for aesthetic purposes in 22 patients. Patients received between 1 and 3 sessions, totaling 39 treatment sessions (Table 2). In several cases, more than one body region was treated in the same session, resulting in a total of 43 treated areas. All patients were women between 30 and 64 years of age (mean 47.0, SD 8.9).

Between 1 and 5 vials of PLLA‐SCA were used per treatment session. Reconstitution occurred immediately before treatment using 14–18 mL of SWFI plus 1–2 mL of lidocaine per 150 mg vial of PLLA‐SCA, depending on the indication. The total volume added ranged between 16 and 20 mL per 150 mg vial of PLLA‐SCA (depending on the indication), resulting in a final PLLA‐SCA concentration of 7.50–9.35 mg/mL (mean 8.35 mg/mL, SD 0.71) (Table 2).

The buttocks were treated in 36% (14/39) of sessions, the neck in 31% (12/39), the abdomen and thighs in 21% (8/39), and the upper arms in 3% (1/39). All body treatments were performed using a 22G blunt cannula, with the application plan being subcutaneous. Specifically for the abdomen, the treatment was applied superficially to Scarpa's fascia.

In three sessions (two on the neck and one on the thighs, 7.69%), patients reported bruising, which appeared on the day of or the day after the procedure and resolved without the need for treatment within 7 days. No nodules or other complications were reported.

### 3.3 Scars

Immediately reconstituted PLLA‐SCA was used for atrophic scar treatment in five patients (three women and two men) who underwent a total of seven sessions (Table 2). The mean age of the group was 38.0 years (SD = 8.4). Scars were treated in the following regions: shoulders, face, neck, suprapubic, and knee.

PLLA‐SCA was reconstituted immediately prior to treatment using a volume of 8–18 mL of SWFI plus 1–2 mL of lidocaine for each 150 mg vial. The total volume ranged from 9 to 20 mL resulting in a final PLLA‐SCA concentration of between 7.50 and 16.67 mg/mL. A reconstituted 150 mg vial of PLLA‐SCA was distributed across an area of approximately 100 cm^2^.

Subcutaneous injection of PLLA‐SCA was performed using a blunt cannula in four treatments, using a needle in one treatment, and using both a needle and a blunt cannula in two treatments (Table 2). In all but one scar treatment, a 2940‐nm fractional erbium laser or radiofrequency microneedling was performed, and the PLLA‐SCA solution was applied immediately afterward.

No adverse events were reported (Table 2).

## 4 Discussion

After its approval in 1999, the recommended reconstitution volume for a vial of PLLA‐SCA has varied from 3 mL of SWFI to 8 mL of SWFI plus 1 mL of lidocaine [10], and the volume increased to 16–20 mL of total solution (including 1 or 2 mL of lidocaine) if used for body treatment rather than for the face or scar tissue [5, 10].

Common adverse events of PLLA‐SCA injections include mild swelling, bruising, and mild pain during the postinjection massages, with complete resolution of these events within the first week after treatment. Subcutaneous nodules have been reported to occur days to several months after PLLA‐SCA injections [11, 12, 13, 17] for several reasons, which include inadequate reconstitution, uneven product distribution, imprecise injection technique, allergic or inflammatory response, or lack of posttreatment massage [2, 8, 9, 18].

Adequate product reconstitution, handling, and injection are central to avoiding many of these adverse events [13]. Although previous research suggested that longer hydration times reduce the risk of nodule formation [18], immediate reconstitution has been recommended by the manufacturer since the beginning of 2021 following in vitro [12] and in vivo [13] research that showed that the immediately reconstituted PLLA‐SCA was safe and effective. Furthermore, a clinical study reported the use of immediately reconstituted PLLA‐SCA in the treatment of cheek wrinkles to be effective and safe [14] and a medical chart review of 4483 treatments in 1002 patients reported nodules only in four patients (0.4%) [15]. Despite these published findings, data involving higher numbers of patients receiving the immediately reconstituted product have been lacking.

Our study corroborates the safety of injection of immediately reconstituted PLLA‐SCA. Solution volumes of 9 mL per vial for facial use and 16–20 mL for body injections have very low reported adverse event rates. In a total of 274 treatments involving immediately reconstituted PLLA‐SCA (as instructed by the manufacturer), only one subcutaneous nodule was reported, and this resolved within 2 weeks following injection of 0.9% saline. Bruising and mild pain were also reported but these are inherent to the procedure, not influenced by the reconstitution method, and resolved without treatment.

Five scars were treated with injectable PLLA‐SCA and through drug delivery using fractional technology (Erbium‐YAG laser). To date, only a few cases [6, 7, 8] of scar treatment with PLLA‐SCA have been reported in the literature, all involving reconstitution 72 h prior to treatment. Consistent with previously reported cases, no nodule formation was observed in our series. While PLLA‐SCA treatment shows promise and appears to be safe for atrophic scars, a larger number of cases is needed to support this affirmation.

In conclusion, the use of immediately reconstituted PLLA‐SCA has been approved since May 2021. While this greatly improves the logistics of treatment for both patients and physicians, many injectors remain unsure about incorporating this change into their routine.

The present study adds to the literature a considerable number of patients treated with the immediately reconstituted PLLA‐SCA as recommended by the manufacturer and reports an adverse event profile similar to that reported with PLLA‐SCA reconstituted 72 h prior to use. This study also provides novel data on the use of PLLA‐SCA with regard to body and scar treatment. The immediate reconstitution and injection of PLLA‐SCA may avoid product loss/wastage and be more time effective for both patients and physicians, without affecting adverse event rates.

A possible limitation of the study is its retrospective nature and the absence of a control group with 72 h prior reconstitution, which could have provided more precise data.

## Author Contributions

All authors have read and approved the final manuscript. R.V.‐B., J.R., F.W., and L.E.T.A. were all involved in the study design, data collection, and analysis. R.V.‐B. led the writing of the manuscript with input from the other authors at all stages of its development.

## Ethics Statement

The project was analyzed and approved for implementation by the Ethics Committee in Switzerland (Ethikkommission Nordwest‐ und Zentralschweiz [EKNZ]) and complied with the laws of the country in which it was performed.

## Conflicts of Interest

Roberta Vasconcelos‐Berg, Julia Real, and Luiz Eduardo Toledo Avelar have received speaker fees from Galderma. Franziska Wenz declares no conflict of interest.

## Data Availability

The data that support the findings of this study are available from the corresponding author upon reasonable request.
